# Mechanism of indoleamine 2, 3‐dioxygenase inhibiting cardiac allograft rejection in mice

**DOI:** 10.1111/jcmm.15024

**Published:** 2020-02-06

**Authors:** Chuan Li, Zhaonan Sun, Fang Yuan, Zhicheng Zhao, Jiehong Zhang, Baotong Zhang, Hongyue Li, Tong Liu, Xiangchen Dai

**Affiliations:** ^1^ Department of General Surgery Tianjin General Surgery Institute Tianjin Medical University General Hospital Heping District China

**Keywords:** allograft rejection, cardiac transplantation, immune tolerance, indoleamine 2,3‐dioxygenase

## Abstract

Indoleamine 2, 3‐dioxygenase (IDO)‐mediated regulation of tryptophan metabolism plays an important role in immune tolerance in transplantation, but it has not been elucidated which mechanism specifically induces the occurrence of immune tolerance. Our study revealed that IDO exerts immunosuppressive effects through two pathways in mouse heart transplantation, ‘tryptophan depletion’ and ‘tryptophan metabolite accumulation’. The synergism between IDO^+^DC and TC (tryptophan catabolic products) has stronger inhibitory effects on T lymphocyte proliferation and mouse heart transplant rejection than the two intervention factors alone, and significantly prolong the survival time of donor‐derived transplanted skin. This work demonstrates that the combination of IDO^+^DC and TC can induce immune tolerance to a greater extent, and reduce the rejection of transplanted organs.

## 1 INTRODUCTION

Organ transplantation is an effective means of clinical treatment of end‐stage organ failure. In addition to the maturity of surgical techniques, the clinical application of immunosuppressive agents such as FK506, CsA and FTY720 has greatly improved the survival rate of organ transplantation. However, these inhibitors do not significantly improve the long‐term survival of the graft, and many problems such as organ transplant rejection and its secondary damage have not been completely solved.^1^ T cell‐mediated immune responses are key factors leading to the rejection of organ transplant. Dendritic cells (DCs) presenting antigens to T cells can lead to two distinct effects, immunogenic or tolerogenic, but the mechanism of tolerogenic effect remains unclear.^2^, ^3^

Activated dendritic cells can express a large amount of indoleamine 2, 3‐dioxygenase (IDO). IDO is the only rate‐limiting enzyme outside the liver that catalyzes the catabolism of tryptophan (TRP) along the kynurenine (KYN) pathway and plays an important regulatory role in transplantation immune tolerance, tumour immune escape, maternal‐foetal immune tolerance and autoimmune diseases.^4^, ^5^ Tryptophan is an essential amino acid for T cell activation and proliferation. Previous studies have shown that high expression of IDO can effectively reduce intracellular tryptophan concentration and down‐regulate T cell immune response, while IDO‐specific inhibitors can significantly induce acute transplant rejection at both the cellular and animal level.^6^, ^7^ Therefore, traditional theory suggests that IDO induces immune tolerance mainly through the tryptophan depletion.

Recent studies have found that the accumulation of tryptophan catabolic products (TC) also plays an important role in transplantation immune tolerance. The main metabolite of IDO decomposition of tryptophan is KYN. Under the action of various enzymes, KYN decomposes into 3‐hydroxyanthranilic acid (3‐HAA), 3‐hydroxykynurenine (3‐HK), anthranilic acid, et cetera.^8^ A study by Brandacher G showed that the severity of postoperative rejection in kidney transplant patients was increased proportionally with the ratio of kynurenine and tryptophan (kyn/trp).^9^ Animal experiments have also shown that tryptophan metabolites such as KYN, 3‐HAA and 3‐HK can significantly inhibit T cell proliferation and prolong the survival time of skin grafts.^10^ Obviously, there are still many controversies about the mechanism by which IDO induces immune tolerance.

In this study, the effects of IDO^+^DC and TC combination on T cell proliferation and apoptosis, cardiac allograft rejection and graft survival were observed at the cellular and animal levels, respectively, then compared with the simple administration of IDO^+^DC or TC.

## 2 MATERIALS AND METHODS

### 2.1 Dendritic cell isolation and culture

Bone marrow cells were collected from C57BL/6 and C3H/He mice (Experimental Animal Center of the Chinese Academy of Military Medical Sciences), respectively, rinsed with PBS and resuspended in RPMI1640 medium containing 10% FBS(Gibco), 10 ng/mL rmGM‐CSF (Perotech) and 2 ng/mL rmIL‐4 (Perotech). Cells were cultured for 72 hours at 37°C in a 5% CO_2_ incubator. Cells were resuspended in RPMI1640 complete medium supplemented with 10 ng/mL rmGM‐CSF, 2 ng/mL rmIL‐4 and 15 ng/mL TNF‐α(Perotech). Then, we adjusted the cell concentration to 1 ~ 2×10^6^/mL and continued to culture for 48 hours in the incubator. All experiments were conducted according to the Chinese Council on Animal Care guidelines of Tianjin Medical University (Batch number: 20130005). This study was approved by the Institutional Animal Ethics Committee of Tianjin Medical University.

### 2.2 IDO adenovirus transfection

Dendritic cells were cultured for 1 day in serum‐free RPMI1640 medium containing 10 ng/mL of rmGM‐CSF and 2 ng/mL of rmIL‐4, and then transfected with IDO recombinant adenoviruses(contained an expression cassette for green‐fluorescent protein [GFP] as a reporter gene; SunBio) with MOI of 10, 50, 100, 200, 250, 300 and 500, respectively. After 2 hours, the complete medium containing cytokines was replaced and continued to culture for 48 hours. Transfection status was observed under a fluorescence microscope (OLYMPUS), and green fluorescence would appear under successful cell transfection. Two days later, GFP expression was quantified by flow cytometry. We observed a dose‐dependent response to the adenoviral infections. At the MOI of 10, 50, 100, 200, 250, 300 and 500, the transfection efficiency was 5%, 10%, 37%, 61%, 69%, 73% or 84%, and the cell survival rate was 100%, 99%, 94%, 91%, 89%, 85% or 76%, respectively. Therefore, the optimal MOI of 200 was selected for the transfection of DCs with AD‐IDO (Ad‐IDO) in this study.

### 2.3 Indoleamine 2, 3‐dioxygenase activity detection (HPLC)

Indoleamine 2, 3‐dioxygenase activity was detected by high‐performance liquid chromatography (HPLC) to quantify the concentration of TRP and KYN in culture medium. C57BL/6 DCs were preincubated with Ad‐IDO or Ad‐Null (Adenoviral vectors without IDO gene). After 48 hours of culture, the different supernatant fluids were collected and mixed with 6% perchloric acid (PCA) at 4:1 ratio, respectively, to precipitate proteins. The acidified samples were centrifuged at 12 000 g at 4°C for 10 minutes, and the supernatant fluids were dried under nitrogen flow. The residues were dissolved in 100 μL of 1% acetonitrile in 0.1% formic acid and transferred to an autosampler vial insert; then, 20 μL of supernatant fluid was injected directly in the high‐performance liquid chromatography system (Alliance separations module 2695; Waters Corp). The chromatographic separation was obtained with an Accucore PFP column (150 mm × 21 mm, 2.6 μm particle size; Thermo‐Scientific) at a flow rate of 0.2 mL/min. Elution started with 99% of mobile phase A (0.1% formic acid in water) and 1% mobile phase B (100% acetonitrile) for 2 minutes, followed by an 18‐minute linear gradient to 50% of phase A, a 1‐minute linear gradient to 30% of phase A and a 1‐minute linear gradient to 99% of phase A, which was maintained for 12 minutes to equilibrate the column. TRP and KYN were detected by UV absorption at 280 nm and 360 nm, respectively (L‐4250 UV VIS Detector, Merck Hitachi).

### 2.4 Mouse spleen CD4^+^ T cell sorting (MACS)

The spleen was taken after the mice were killed, and the precipitated cells were collected after grinding, filtration and centrifugation. The red blood cells were completely lysed by adding red blood cell lysate (BD), and the cell pellet was rinsed twice with PBS after centrifugation. The cells were resuspended in pre‐cooled binding buffer, and then the CD4 immunomagnetic beads (Miltenyi) were added and incubated for 15 minutes at 4°C. After the incubation, the cells were rinsed and resuspended in binding buffer. The cell suspension was added to the MS column (Miltenyi) until it was drained, and the column was washed three times with the washing buffer. The rinse solution was collected when rinsing the MS column with the elution buffer.

### 2.5 T lymphocyte proliferation assay (MTS)

One‐way mixed lymphocyte reactions (MLR) were performed in duplicate on 96‐well tissue culture plates (Asahi Techno Glass) by using responder CD4^+^ T cells and stimulator DCs. In addition, BALB/c mice DCs were harvested as negative control, and mix‐cultivated with homologous CD4^+^ T cells. Various cells were harvested as previously described. Allogeneic CD4^+^ T cells (1 × 10^6^/mL) were plated with different groups at a ratio of 10:1 (DCs at a concentration of 1 × 10^5^/mL). After incubation at 37°C in a humidified incubator with 5% CO_2_ for 48 hours, cells were exposed to 20 μL of 3‐(4,5‐dimethylthiazol‐2‐yl)‐5‐(3‐carboxymethoxyphenyl)‐2‐(4‐sulfophenyl)‐2H‐tetrazolium inner salt (MTS) and cultured for 4 hours according to the manufacturer's instructions (Promega Corporation). MTS is bioreduced by cells into a coloured formazan product that reduces absorbance at 490 nm. The optical density (OD) was read at a wavelength of 490 nm with an iMark Microplate Absorbance Reader (Bio‐Rad). The mean OD value calculated with triplicate wells was used in the following formula: T cell proliferation stimulation index (SI) = (OD_experimental group_ − OD_culture solution_)/(OD_negative control_ − OD_culture solution_).

### 2.6 T cell apoptosis assay (Annexin‐FITC/PI)

Detection of early apoptotic CD4^+^ T cells was performed using the annexin V and propidium iodide (PI) detection kit (BD Biosciences). CD4^+^ T cells were harvested after mixed lymphocyte reactions with different DCs as described above. Briefly, 10^6^ isolated T cells were washed once in PBS, incubated in the dark at 4°C with annexin V‐fluorescein isothiocyanate (annexin V–FITC) and PI for 15 minutes in 500 μL 1× binding buffer and then analysed by dual‐colour flow cytometry within 1 hour. Cells that were annexin V‐FITC positive (indicating translocation of phosphatidylserine from the inner to the outer leaflet of the plasma membrane), and PI negative (with intact cellular membrane) were considered as early apoptotic cells.

### 2.7 Haematoxylin staining and Immunohistochemistry

Heart graft samples were collected at 7 days after transplantation and fixed in 10% buffered formaldehyde, embedded in paraffin and sectioned at 5 μm for haematoxylin (HE) staining. The ensuing morphological examination was performed using an Olympus Microscope (X51). Criteria for graft rejection included the presence of lymphocyte infiltration and interstitial oedema. Briefly, IHC was used on 5 μm sections (Leica) taken from ice‐cold 4% (w/v) paraformaldehyde‐fixed paraffin‐embedded tissue. Sections were heated at 60°C for 1 hour, then deparaffinized in three times of xylene and rehydrated with graded concentrations of ethanol. For immunostaining (BOSTER), deparaffinized and rehydrated sections were heated in citrate buffer at 121°C for 30 minutes and incubated with 0.3% hydrogen peroxide in methanol for 20 minutes, respectively. After non‐specific reactions had been blocked with 5% BSA (Solarbio), the sections were incubated with primary antibody at 4°C overnight and then with biotinylated secondary antibody at 37°C for 1 hour. The sections were counterstained with haematoxylin for detection.

### 2.8 RNA Isolation and RT‐PCR Analyses

The total RNA was isolated using RNA extraction kit (TIANGEN) and reverse transcribed into cDNA using reverse transcription kit (ABI). Quantitative real‐time PCR analysis was performed using real‐time PCR kit (ABI). The relative mRNA expression levels of IDO, IL‐2, IFN‐γ, IL‐10, IL‐4 and TNF‐αwere normalized with the β‐actin in the same sample. The thermal cycler parameters for the amplification of these genes were as follows: 1 cycle at 95°C for 10 minutes followed by 40 cycles at 95°C for 15 seconds, 60°C for 15 seconds and 72°C for 30 seconds. Gene expression was evaluated by the 2^−ΔΔCt^ method. The sequences of RT‐PCR primers are the following (5′‐3′, Table 1).

**Table 1:** The sequences of RT‐PCR primers

	Forward	Reverse
IL‐2	GGCATGTTCTGGATTTGACTC	CTCATCATCGAATTGGCACTC
IFN‐γ	ATGAACGCTACACACTGCATC	CCATCCTTTTGCCAGTTCCTC
IL‐10	AGAAGCATGGCCCAGAAATCA	GGCCTTGTAGACACCTTGGT
TNF‐α	CATCTTCTCAAAATTCGAGTGACAA	TGGGAGTAGACAAGGTACAACCC
IL‐4	ACAGGAGAAGGGACGCCAT	GAAGCCCTACAGACGAGCTCA
IDO	CAGAGCAGCATCTTCCAGAGT	CCAGACCATTCACACACTCGT
β‐actin	ATCCGTAAAGACCTCTATGC	AACGCAGCTCAGTAACAGTC

### 2.9 Western blot

Total protein was extracted from donor heart tissues (Qiagen DNeasy kit; Qiagen) according to the manufacturer's instructions. Proteins (20 μg of total protein) were subsequently separated by SDSPAGE, and then transferred to PVDF membranes. The membranes were blocked with 5% skimmed milk in Tris‐buffered saline with Tween‐20 (TBST) at room temperature for 2 hours. Then, the proteins were incubated overnight with rabbit antimouse IDO (Abcam; 1:200) at 4°C. The membranes were then incubated with HRP‐conjugated goat anti‐rabbit second antibody (Abbiotec; 1:1000) for 1 hour at room temperature. After washing the membrane thrice with TBST, the proteins were visualized using an ECL detection system (G: BOX, Syngene). GAPDH (Abbiotec; 1:1000) was used as the internal control. The density of each band was determined using the corresponding GAPDH value. Bands were scanned and quantitated by densitometry using Quantity one imaging software.

### 2.10 Construction of a mouse allogeneic heart transplantation model

Briefly, a midline abdominal incision was performed on the donor. The inferior vena cava, the superior vena cava and azygous vein were ligated and divided. The ascending aorta and pulmonary artery were separated and divided distally as far as possible. The pulmonary veins were ligated together, and then the graft was removed from the donor. The proximal and distal ligatures were placed in the recipient around the aorta and inferior vena cava, respectively. The donor's heart was placed on the right side of the recipient's abdomen. The stay sutures were placed at the proximal and distal apices of the recipient's abdominal aorta with the donor's ascending aorta. The anastomosis of the recipient's abdominal aorta and the donor's ascending aorta was closed with continuing sutures. The anastomosis of the donor's pulmonary artery and the recipient's inferior vena cava was made in a similar fashion. After the ligatures were removed, the colour of the heart became red and it began to beat again. The mice were given dextrose saline solution and conventional feeding after woken, and 4000 U/kg amikacin was injected for 3 days. The standard for successful surgery is that the heartbeat lasts for at least 3 days. There were twelve recipients in each group. Six recipients in each group were used to observe the donor's survival time, and the remaining six were killed at 7 days post‐transplantation for HE staining, real‐time PCR and Western blot.

### 2.11 Establishment of mouse skin transplantation model

Mice were fixed in prone position after anaesthesia. 1 cm^2^ of the skin on the back of the donor was taken off after disinfection. The same size piece of skin was cut from the same part of the receptor. Insert the donor skin in the direction of the reverse hair and make it consistent.

### 2.12 Statistical analysis

Data were compared and reported as mean ± standard deviation. Statistical comparison between various groups was performed by one‐way analysis of variance with Tukey's post hoc adjustment for multiple comparisons, as appropriate, using the SPSS software (SPSS, Inc). Allograft survival among experimental groups was compared using log‐rank (Mantel‐Cox) testing. Differences were considered statistically significant when *P* value was <.05.

## 3 RESULTS

### 3.1 Combined application of IDO + DC and TC has a stronger inhibitory effect on T lymphocytes than the two intervention factors alone

**Figure 1:**
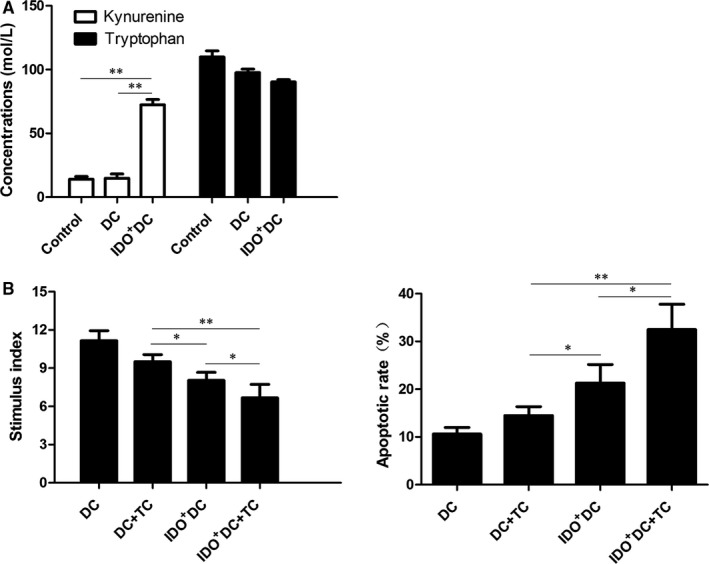
Combined application of IDO^+^DC and TC has a stronger inhibitory effect on T lymphocytes in vitro than the two intervention factors alone. A, Concentrations of kynurenine and tryptophan in each group. AD‐IDO was transfected into DC cell for 48 h. Cell supernatant was added to a final concentration of 4% trichloroacetic acid, and the concentrations of tryptophan and kynurenine were measured by HPLC. The kynurenine concentration was significantly higher in the IDO^+^DC group compared with the DC and control groups, while not significantly different between the control and DC group, respectively, indicating that the IDO gene was actively expressed after transfection. B, CD4^+^ T cells were mix‐cultivated with the different DCs. Annexin V and PI double staining was performed to detect the apoptosis of T cells in DC, DC + TC, IDO^+^DC and IDO^+^DC+TC groups. T cells apoptosis rate in IDO^+^DC+TC group was significantly higher compared with other groups. T cells proliferation stimulation index in IDO^+^DC+TC group was significantly lower compared with other groups. It indicated that CD4^+^ T cells suppression was significantly stronger in IDO^+^DC+TC group in vitro. Data are shown as mean ± SD for three independent experiments; *P < .05, **P < .01

Previous studies have found that there is a Tolerogenic DC (Tol‐DC) that lacks costimulatory function and exhibits high IDO activity in long‐lived heart transplant model receptors.^11^ Because of the small proportion of Tol‐DC, we cultured mouse bone marrow‐derived DC in vitro, and transfected the IDO gene into DC by recombinant adenovirus. Cells were then cultured with different intervention factors in vitro and in vivo, respectively. The activity of IDO after transfection was detected by HPLC. Results showed that the concentration of kynurenine in the IDO^+^DC group was significantly higher than that in the control group and the DC group (72.462 ± 4.083 mM vs 14.026 ± 2.186 and 14.766 ± 3.447 mM, respectively, *P* < .01), suggesting that the transfected DC cells have higher IDO activity (Figure 1A). The decrease of tryptophan concentration in the IDO^+^DC group was not significant, probably because the initial concentration of tryptophan in the medium was much higher than the number of IDO^+^DC cells.

**Figure 2:**
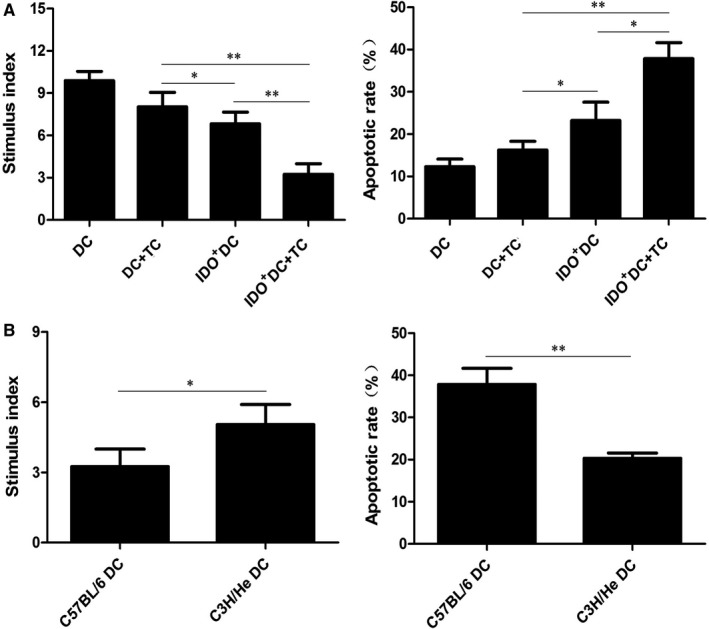
Combined application of IDO^+^DC and TC has a stronger inhibitory effect on T lymphocytes and this effect may be antigen‐specific in vivo. A, BALB/c recipients were randomly assigned to four groups: DC group (1 × 106 donor's untreated DCs, intravenously, on days − 3, −1); DC + TC group (1 × 106 donor's untreated DCs and 200 uM TC, intravenously, on days − 3, −1); IDO^+^DC group (1 × 106 donor's DCs transfected by Ad‐IDO, intravenously, on days − 3, −1); IDO^+^DC+TC group (1 × 106 donor's DCs transfected by Ad‐IDO and 200 uM TC, intravenously, on days − 3, −1). Annexin V and PI double staining was performed to detect the apoptosis of T cells in each group. T cells apoptosis rate in IDO^+^DC+TC group was significantly higher compared with other groups. T cells proliferation stimulation index in IDO^+^DC+TC group was significantly lower compared with other groups. It indicated that CD4^+^ T cells suppression was also significantly stronger in IDO^+^DC+TC group in vivo. (B) Flow cytology analysis of different sources of DC stimulation on T cell apoptosis and proliferation. C3H/He‐derived DC had higher T cell stimulation index and lower T cell apoptosis rate than C57BL/6‐derived DC. Data are shown as mean ± SD for three independent experiments; *P < .05, **P < .01

In vitro experiments showed that the T cell stimulation index of DC + TC, IDO^+^DC, IDO^+^DC+TC group was significantly lower than that of DC group (9.506 ± 0.568, 8.042 ± 0.625 and 6.673 ± 1.053 vs 11.154 ± 0.791, respectively, *P* < .05), while the apoptosis rate of T cell was increased (14.467 ± 1.898, 21.267 ± 3.901 and 32.467 ± 5.302 vs 10.633 ± 1.340, respectively, *P* < .05). Besides, compared with IDO^+^DC and TC groups, the decrease of T cell stimulation index and the degree of T cell apoptosis were more pronounced in the IDO^+^DC+TC group (*P* < .01, Figure 1B). This result was also verified in vivo experiments in which donor DCs were C57BL/6 mice (Figure 2A), indicated that IDO inhibited T cell proliferation through both ‘tryptophan depletion’ and ‘tryptophan metabolite accumulation’ pathways.

To investigate the effects of different sources of DC stimulation on T cell proliferation, we introduced a tertiary DC (C3H/He) to perform experiments under the same conditions. Results showed that C3H/He‐derived DC had higher T cell stimulation index and lower T cell apoptosis rate than C57BL/6‐derived DC (5.046 ± 0.851 vs 3.247 ± 0.744, *P* < .05 and 20.301 ± 1.252 vs 37.834 ± 3.799, *P* < .01, Figure 2B). This suggested that the combination of IDO^+^DC and TC might induce the occurrence of immune tolerance in vivo, but it still needed to be further explored.

### 3.2 Combined application of IDO^+^DC and TC has a stronger inhibitory effect on cardiac allograft rejection in mice than the two intervention factors alone

**Figure 3:**
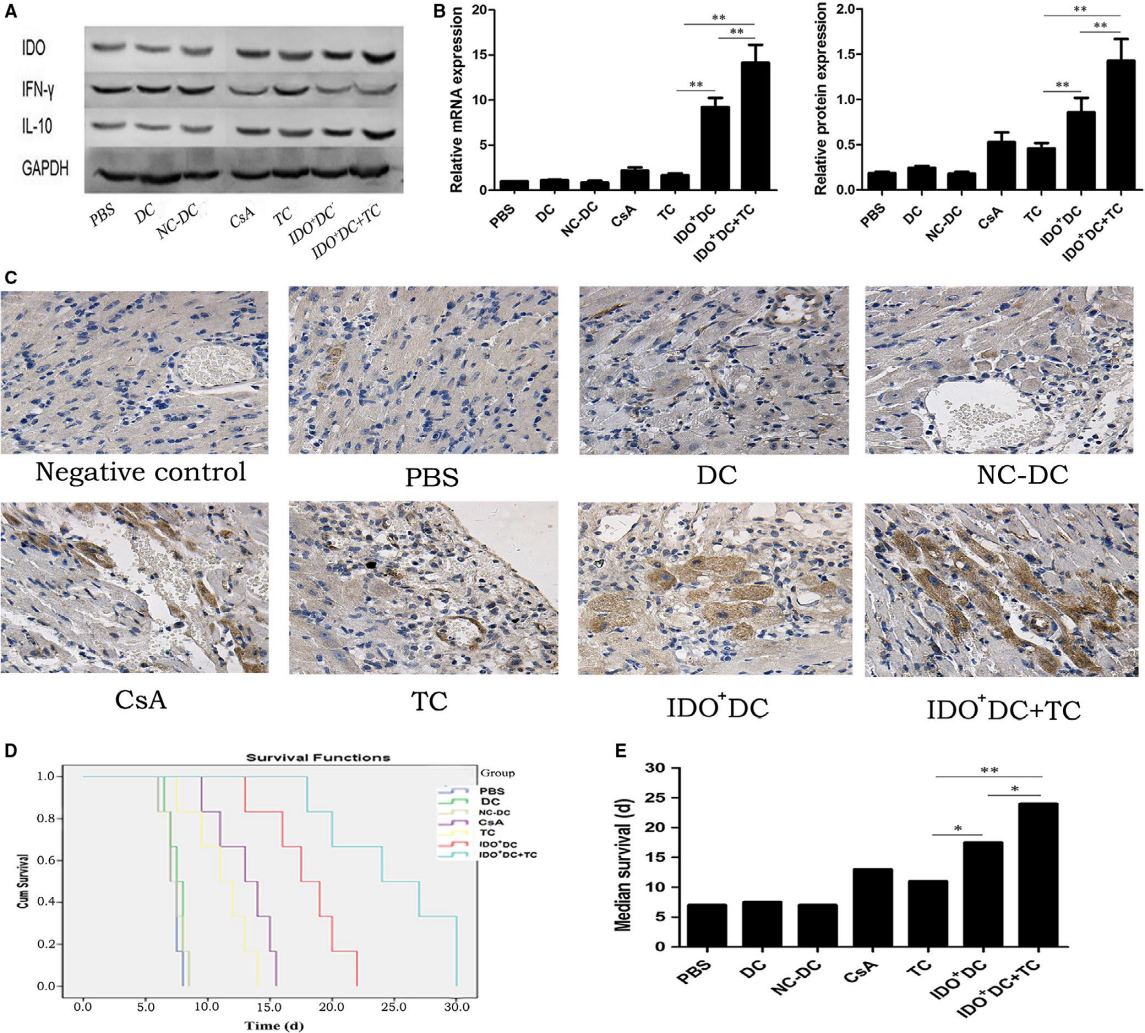
Combined application of IDO^+^DC and TC has a stronger inhibitory effect on cardiac allograft rejection in mice than the two intervention factors alone. BALB/c recipients were randomly assigned to seven groups: PBS group (PBS, intravenously, on days −3, −1); DC group (1 × 106 donor's untreated DCs, intravenously, on days −3, −1); NC‐DC group (1 × 106 donor's DCs transfected by Ad‐Null, intravenously, on days −3, −1); CsA group (CsA 10 mg/kg/d, intraperitoneal, on days 1 − 5); TC group (200 μM TC, intravenously, on days− 3, −1); IDO^+^DC group (1 × 106 donor's DCs transfected by Ad‐IDO, intravenously, on days −3, −1); IDO^+^DC+TC group (1 × 106 donor's DCs transfected by Ad‐IDO and 200 uM TC, intravenously, on days −3, −1). A, The protein expression of IDO, IFN‐γ and IL‐10. Total protein of donor heart tissues in each group was extracted and then subjected to Western blot. GAPDH was used as the loading control. B, Quantification of IDO mRNA and protein. Total RNA and protein were extracted from heart tissues and then subjected to RT‐PCR and Western blot. The ratio of IDO/GAPDH in the IDO^+^DC+TC group was significantly higher compared with other groups, which indicated that combined application of IDO^+^DC and TC make IDO highly expressed in donor hearts than the two intervention factors alone after transplantation. C, Immunohistochemical staining of IDO in transplanted hearts (×400). The expression of IDO protein in the IDO^+^DC+TC group was significantly higher compared with TC and IDO^+^DC groups, respectively. Furthermore, in the donor hearts, IDO was mainly expressed in heart muscle cells and also weakly stained in blood vessels. D, Kaplan‐Meier analysis of transplanted heart survival in each group. E, Quantification of median survival in (D). Data are shown as mean ± SD for six independent experiments; *P < .05, **P < .01

To further study the effect of IDO on animal organ transplant rejection, we infected the mature DC with IDO‐bearing adenovirus in vitro to obtain Tol‐DC. IDO^+^DCs were injected into Balb/C mice via the tail vein, and allogeneic heart transplantation was performed three days later. The mRNA and protein expression of IDO in each groups were detected by RT‐PCR, Western blot and immunohistochemistry. The mRNA expressions in the seven groups of PBS, DC, NC‐DC, CsA, TC, IDO^+^DC and IDO^+^DC+TC were 1, 1.127 ± 0.063, 0.859 ± 0.201, 2.179 ± 0.371, 1.678 ± 0.217, 9.238 ± 1.006 and 14.136 ± 1.989, respectively. In addition, the protein expressions were 0.185 ± 0.017, 0.245 ± 0.021, 0.180 ± 0.020, 0.529 ± 0.108, 0.460 ± 0.057, 0.859 ± 0.158 and 1.429 ± 0.240, respectively. Results indicated that the mRNA and protein expressions of IDO in the CsA group, the TC group, the IDO^+^DC group, and the IDO^+^DC+TC group were significantly higher than those in the PBS group, the untransfected DC group, and the DC transfected with empty virus (NC‐DC) group, and the increase was more obvious in the IDO^+^DC+TC group (Figure 3A,B). Immunohistochemical staining showed that IDO protein was mainly expressed in the cytoplasm of the cardiomyocytes and the vessel wall. The positive rate and staining intensity of IDO in the CsA group, the TC group, the IDO^+^DC group and the IDO^+^DC+TC group were significantly higher than other groups, and it was most significant in the IDO^+^DC+TC group (Figure 3C). It was suggested that the IDO overexpressing mouse heterotopic heart transplantation model was successfully constructed. Kaplan‐Meier survival analysis showed that the survival time of the transplanted hearts in the TC group and the IDO^+^DC group was significantly longer than that in the PBS group and the DC group (Figure 3D). Moreover, the median survival time of the transplanted hearts in the IDO^+^DC+TC group was significantly longer than that in the TC group and the IDO^+^DC group (24 days vs 11 and 17.5 days, respectively, *P* < .01, Figure 3E).

**Figure 4:**
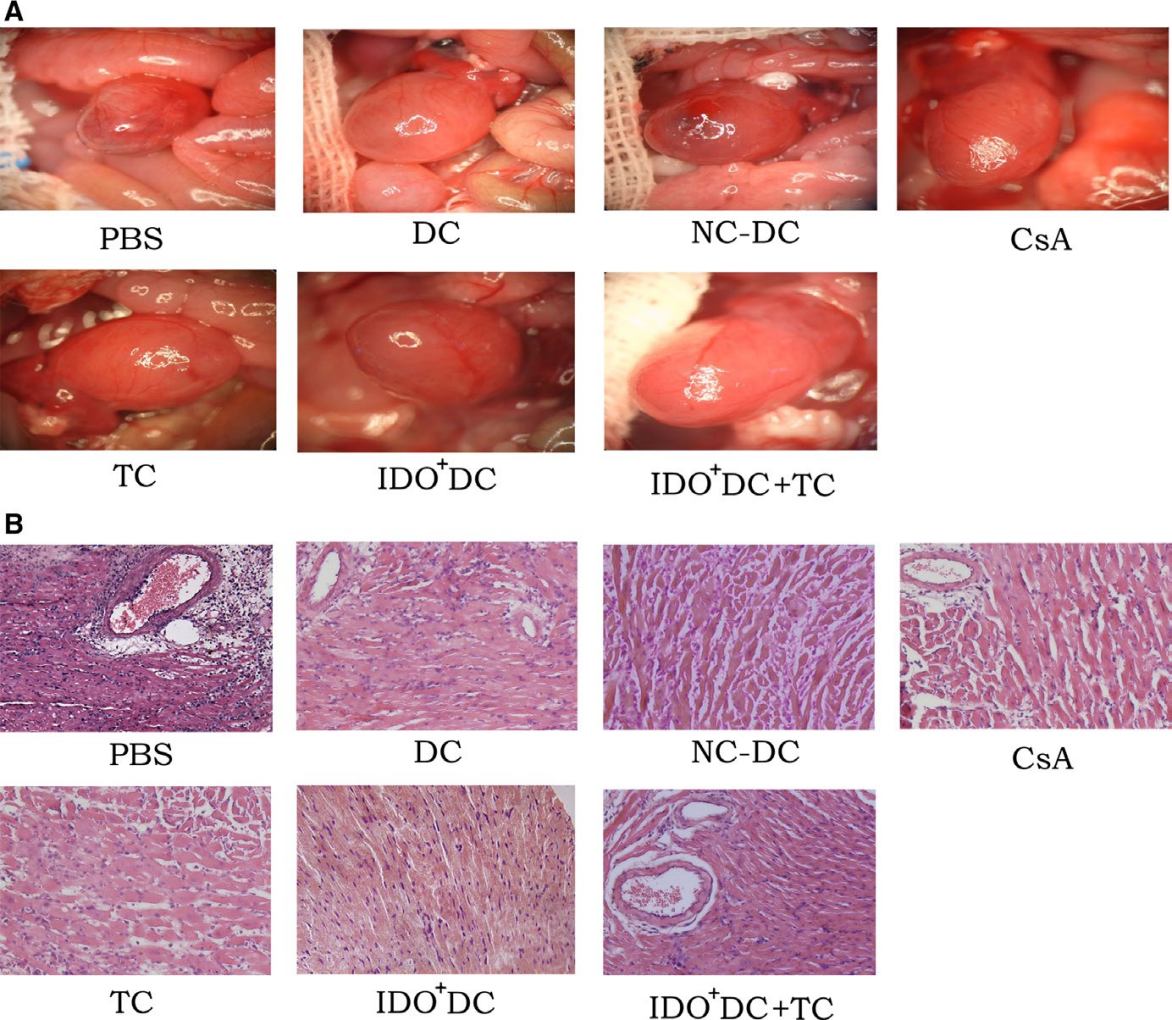
General and microscopic appearance of donor heart. A, The phenotype of transplanted heart in each group. B, Histopathological manifestations of transplanted heart in each group (×200). Transplanted hearts were harvested at postoperative day 7 and performed with haematoxylin‐eosin staining and then observed under an optical microscope. Heart grafts were rejected with severe cellular infiltration and interstitial oedema in PBS, DC and NC‐DC group. There was only mild inflammatory cells infiltration of the heart grafts in CsA, TC and IDO^+^DC group. In contrast, almost normal histology is apparent in the IDO^+^DC+TC group

In addition, the phenotype and recovery status of transplanted hearts in the CsA group, the TC group, the IDO^+^DC group and the IDO^+^DC+TC group were much better than control groups (Figure 4A). Histopathological examination showed multifocal inflammatory cell infiltration and severe myocardial damage in the PBS group and the untransfected DC group manifested as moderate‐to‐severe acute rejection. While the cardiomyocytes in the IDO^+^DC group had slight focal lesions and a small amount of inflammatory cell infiltration, which showed mild rejection. In contrast, the degree of myocardial damage was even weaker in the IDO^+^DC+TC group, and allogeneic rejection was more significantly inhibited (Figure 4B and Table 2).

**Table 2:** Comparison of pathological grades of transplanted hearts (n = 6)

Group	pathological grade				*χ^2^*	*P*
	Grade 0	Grade 1	Grade 2	Grade 3		
PBS	0	0	0	6	30.563	.000
DC	0	0	1	5		
NC‐DC	0	0	0	6		
CsA	0	3	2	1		
TC	0	2	3	1		
IDO^+^DC	1	3	2	0		
IDO^+^DC+TC	3	2	1	0		

Note

Kruskal Wallis Test.

**Figure 5:**
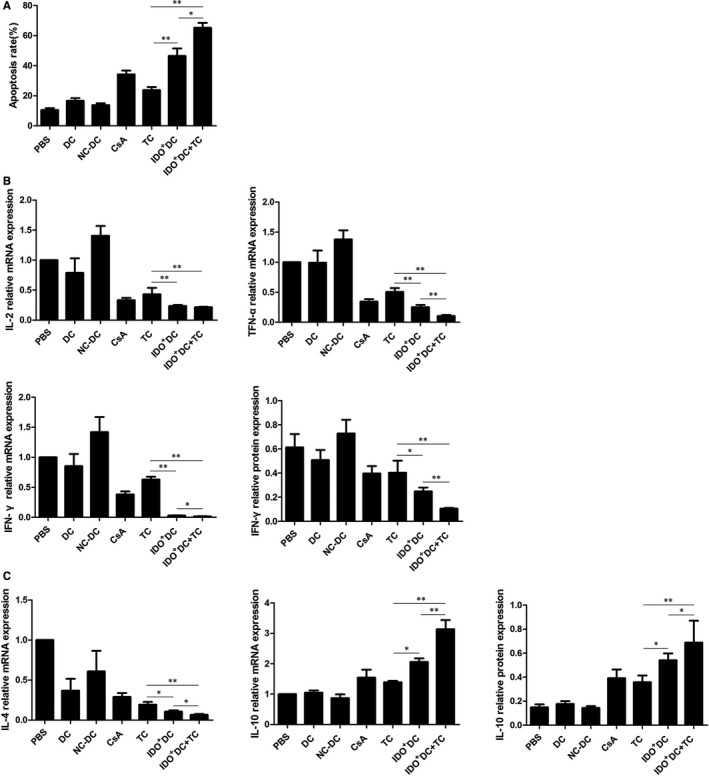
Combined application of IDO^+^DC and TC promote Th2 cytokine shift. A, Annexin V and PI double staining was performed to detect the apoptosis of T cells in each groups. CD4^+^ T cells were harvested from peripheral blood of recipients at 7 d after cardiac transplantation. T cells apoptosis rate in IDO^+^DC+TC group was significantly higher compared with TC and IDO^+^DC groups. B, Expression of Th1‐related cytokine (IL‐2, TFN‐α, IFN‐γ). Total RNA and protein were extracted from heart tissues and then subjected to RT‐PCR and Western blot. The IL‐2, TFN‐α and IFN‐γ level in IDO^+^DC+TC group was significantly lower compared with TC and IDO^+^DC groups. C, Expression of Th2‐related cytokine (IL‐4, IL‐10). Total RNA and protein were extracted from heart tissues and then subjected to RT‐PCR and western blot. The IL‐4 and IL‐10 level in IDO^+^DC+TC group was significantly higher compared with TC and IDO^+^DC groups, indicating that IDO^+^DC+TC shift cytokine towards a Th2‐dominant response. Data are shown as mean ± SD for six independent experiments; *P < .05, **P < .01

The apoptosis rates of CD4^+^ T cells in PBS, DC, NC‐DC, CsA, TC, IDO^+^DC and IDO^+^DC+TC group were 10.633 ± 1.221, 16.733 ± 1.749, 13.833 ± 1.201, 34.333 ± 2.429, 23.767 ± 2.089, 46.500 ± 5.022 and 65.233 ± 3.289 (%), respectively, as measured by flow cytometry. This result showed that the apoptosis rate of receptor T cells was significantly higher than that of the control group after IDO^+^DC and TC intervention, and the degree of apoptosis was more obvious in the IDO^+^DC+TC group (*P* < .01, Figure 5A). All of the above data indicated that the combined effect of IDO^+^DC and TC could significantly increase the survival time of transplanted hearts in mice, and induced the apoptosis of T cells, thereby inhibiting rejection of transplanted hearts. However, the specific inhibition mechanism of IDO on the immune system remains unclear.

Studies have shown that DCs with high expression of IDO can significantly down‐regulate Th1‐mediated immune responses, while inhibitors of IDO can aggravate Th1‐mediated immune responses by inhibiting IDO activity.^11^, ^12^ IDO and its metabolites induce apoptosis in Th1 cells, but the effect on Th2 cells is unclear.^13^ Th1/Th2 balance plays a crucial role in maintaining the body's immune homeostasis.^14^ To further clarify the mechanism by which IDO induces immune tolerance, we examined the expression of Th1 and Th2‐related cytokines. RT‐PCR and Western blot showed that compared with the control group, the mRNA and protein expression of IL‐2, IFN‐γ and TFN‐α were significantly decreased after treatment with IDO^+^DC and TC, while the mRNA and protein expression of IL‐10 were significantly elevated, and the changes of all the above‐mentioned cytokines in the IDO^+^DC+TC group were more significant than those in the TC group and the IDO^+^DC group (Figures 3A, 5B,C). These results suggested that there was a close relationship between the shift of Th1/Th2 balance and transplant rejection. IDO^+^DC and TC administration skewed the Th1/Th2 balance towards Th2 dominance, thereby inhibiting mouse heart transplant rejection more effectively.

### 3.3 Combined application of IDO + DC and TC can significantly prolong the survival time of donor‐derived transplanted skin

**Figure 6:**
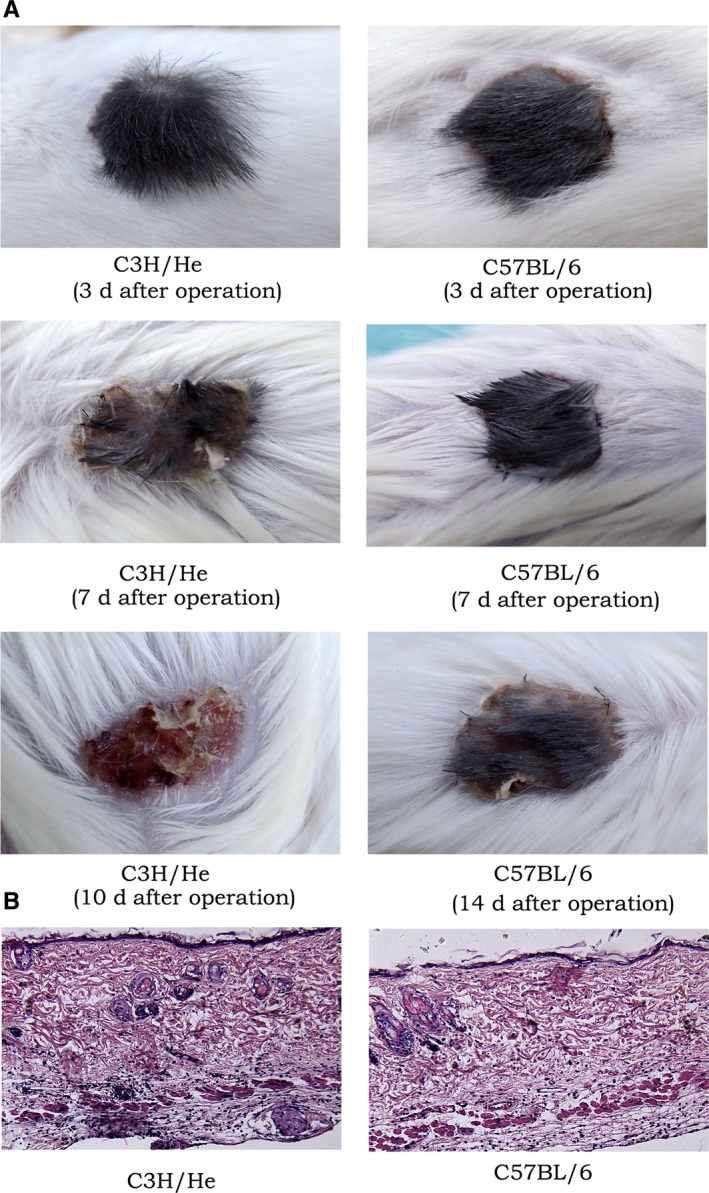
Combined application of IDO^+^DC and TC can significantly prolong the survival time of donor‐derived transplanted skin. A, General condition of transplanted skin at different time after surgery in the C3H/He and C57BL/6 groups. B, Pathological observation of transplanted skin 7 d after operation (×200). Transplanted skin was performed with haematoxylin‐eosin staining and then observed under an optical microscope

To further clarify whether IDO^+^DC+TC has specific immunosuppression against donor antigens, we re‐established the model according to the processing method of the IDO^+^DC+TC group in the previous section. Mice with heart transplantation survived for more than 30 days were randomly divided into two groups, skin grafts of donor source (C57BL/6) and tertiary source (C3H/He) were performed separately. The survival of transplanted skin in each group was observed, and pathological examination of skin grafts was performed by immunohistochemistry. Results showed that the transplanted skin of C57BL/6 originated well with clear tissue hierarchy and vascular structure. Fourteen days after surgery, the transplanted skin showed mild scarring and hair loss, and only a small amount of inflammatory cells infiltrated in the subcutaneous tissue (Figure 6A and 6). However, the C3H/He‐derived transplanted skin showed rejection only 1 week after surgery, with dispersed tissue and vascular, and the subcutaneous tissue was accompanied by a large amount of inflammatory cell infiltration. The transplanted skin was completely scarred and shedding 10 days after surgery (Figure 6A and 6). The above results indicated that IDO^+^DC and TC interventions have differential immune defenses for different sources of stimulation. It was suggested that the combination of IDO^+^DC and TC could induce the occurrence of immune tolerance to a certain extent, but its long‐term effect and specific mechanism still needed to be further explored.

## 4 DISCUSSION

Previous studies have shown that IDO may exert immunosuppressive function through two different pathways, ‘tryptophan depletion’ and ‘tryptophan metabolite accumulation’, but most studies only explored from a single contributing factor. This work complemented and integrated traditional theories at the cellular and animal levels by combining two mechanisms. At the cellular level, we demonstrated that the combination of IDO^+^DC and TC significantly inhibits the proliferation of T cells, thereby inducing antigen‐specific immunosuppression. The establishment of specific immune tolerance is of great value for clinical transplantation. Although non‐specific immunosuppressive agents have been widely used in the clinic, patients often suffer from various comorbidities due to low immune function, so the effective establishment of transplant immune tolerance has become the top priority of clinical transplantation.^15^, ^16^ Therefore, we further studied the immunosuppressive effects of IDO^+^DC and TC in animal level by establishing mouse allogeneic heart transplantation model. Results showed that the combination of IDO^+^DC and TC could significantly prolong the survival time of transplanted hearts, reduce the extent of transplant rejection and induce apoptosis of recipient T cells, which might be related to the inhibition of the release of Th1 cytokines and the promotion of differentiation of native CD4^+^ T cells into Th2 subsets. Therefore, we speculated that IDO exerts its immunosuppressive effects on both the ‘tryptophan depletion’ and ‘tryptophan metabolite accumulation’ pathways. Skin transplantation experiments showed that the survival time of donor‐derived skin was greatly prolonged compared with the tertiary‐derived skin, and the pathological degree of rejection was much lower, suggesting that IDO treatment before organ transplantation is expected to induce transplantation tolerance.

Indoleamine 2, 3‐dioxygenase was originally considered to be one of the effect mechanisms of resistance to infection due to its high expression in inflammation. With the deepening of research, IDO has been found to play an important role in maternal‐foetal immune tolerance, tumour immune escape and regulation of transplant immune tolerance.^17^, ^18^ Current research on IDO‐mediated immunoregulatory mechanisms involves the following processes: Overexpression of IDO can lead to a decrease in local tryptophan content, an increase in kynurenine and other metabolites. Decreased tryptophan concentration can inhibit the activity of immune killer cells such as CD4^+^ effector T cells and NK cells, and promote their apoptosis.^19^ Furthermore, changes in tryptophan and metabolite concentration can also induce differentiation of tolerogenic DC (Tol‐DC) and promote proliferation and activation of immune negative regulatory cells such as Treg cells.^20^, ^21^ The above factors collectively participate in the induction of immune tolerance. Our study confirmed that IDO corporately induces cardiac allograft immune tolerance in mice through ‘tryptophan depletion’ and ‘kynurenine accumulation’, which may provide new ideas and methods for clinical induction of transplant immune tolerance.

## CONFLICT OF INTEREST

The authors declare that they have no competing interests.

## AUTHOR CONTRIBUTIONS

Chuan Li, Tong Liu, Xiangchen Dai conceptualized and designed the study. Chuan Li, Zhaonan Sun, Fang Yuan written, reviewed and revised the manuscript. Chuan Li, Zhaonan Sun, Zhicheng Zhao and Jiehong Zhang developed the methodology. Chuan Li, Fang Yuan, Baotong Zhang and Hongyue Li analysed and interpreted the data. Tong Liu and Xiangchen Dai supervised the study.

## Data Availability

The data that support the finding of this study are available from the corresponding author upon reasonable request.
